# Sport training day affects adolescent athletes’ sleep schedules

**DOI:** 10.3389/fspor.2025.1731173

**Published:** 2026-01-27

**Authors:** Abigail Larson, Jena Heck Street, Roman de Guia, Jacob J. Capin

**Affiliations:** 1 Life After Sport Trajectories (LAST) Lab, Department of Physical Therapy, Marquette University, Milwaukee, WI, United States; 2 Clinical and Translational Science Institute of Southeast Wisconsin, Medical College of Wisconsin, Milwaukee, WI, United States

**Keywords:** accelerometry, adolescent, athlete, high school, sleep, sports, training day

## Abstract

**Introduction:**

Sufficient sleep is integral to overall health whereas suboptimal sleep puts athletes at greater risk for injury, lengthens their recovery time, and decreases their performance. Our purpose was to compare sleep across training days (i.e., competition, practice, and rest) in adolescent athletes, specifically identifying differences between nights before and after training.

**Methods:**

Participants [*n* = 33 (14 male, 19 female), aged 15.6 ± 1.0 years] wore an activPAL accelerometer continuously for 14 consecutive days and completed a sleep log noting training day status (i.e., competition, practice, rest).

**Results:**

Athletes slept less on the day after competition compared to after practice and rest days [Sleep Duration (mean ± SD) after competition: 7.9 ± 1.6 h, after practice: 8.6 ± 0.8 h, after rest: 9.2 ± 1.4 h, interaction effect *p* < 0.001]. The time athletes went to bed tended to be later before and after rest days compared with competition and practice days [Time in Bed Start (hr:min, mean ± SD) before competition: 22:02 ± 0:58, after competition: 22:19 ± 1:03, before practice: 22:20 ± 0:49, after practice: 22:06 ± 0:40, before rest: 22:55 ± 1:09, after rest: 23:13 ± 0:58, *p* = 0.080]. Athletes woke up the latest on and after rest days (before competition: 6:52 ± 1:16, after competition: 6:11 ± 1:21, before practice: 6:59 ± 0:43, after practice: 6:43 ± 0:42, before rest: 7:35 ± 1:09, after rest: 8:26 ± 1:22, *p* < 0.001).

**Conclusion:**

In conclusion, athletes slept less the day after a competition compared with the day after a practice or rest day. While sleep start times did not differ by training day, athletes woke up later before and after rest days. Understanding factors influencing athletes’ sleep may enable health care providers and coaches to help athletes stay healthy and optimize their performance.

## 1 Introduction

Sleep is an integral part of human health, directly affecting cognitive function, physical recovery, and physiological status (1–3), all of which impact athletic performance. Therefore, sleep should be a vital part of an athlete's life and schedule. Many people, including athletes, do not meet the recommended sleep guidelines (4, 5). Insufficient sleep can impair performance, as less than 7 h of sleep the night prior to testing results in significantly longer gait times, indicating a decrease in motor skills (6). Another study reported that reduced sleep decreases comprehension and recall of training and game strategies, increases errors in high pressure situations, and decreases reaction time (7). Longer sleep times facilitate tennis serve accuracy in college varsity tennis players (8, 9) and shot percentage in collegiate basketball athletes (10). Evaluating sleep quantity and sleep schedules in athletes may therefore be a critical factor to consider when prioritizing performance and recovery.

When comparing sleep duration between athletes and non-athletes, there are conflicting findings, and considerably more research has been in adult (college and elite) athletes rather than adolescent athletes. Some studies report insufficient sleep duration in athletes (7, 11) while others suggest athletes get the recommended hours of sleep (12, 13). Athletes’ schedules are very busy; therefore their sleep schedules may change based on the time of training and travel, training day, and other obligations (14). Large individual variability has been identified in elite athletes’ sleep schedules; this variability may negatively affect health and performance (15). Training day affects sleep in elite athletes, as sleep duration was shorter immediately after increased training load, specifically on competition days and hard training days (16, 17). Sleep onset and offset in elite athletes were earlier and sleep duration was shorter the night of a training day compared to the night of a rest day (14). Shorter sleep duration may be associated with earlier training start times: in both elite and adolescent athletes, as training time is pushed earlier, sleep duration decreases (14, 18).

Adolescence is a critical age of development during which sufficient sleep is essential. The American Academy of Sleep Medicine recommends that adolescents aged 13–18 years should sleep at least 8 h per 24-hour day (10). Approximately 27.3 million adolescents, aged 6–17 years old, participated in a team sport in the United States during the year of 2023 (19). Adolescent athletes’ schedules and training loads are becoming increasingly more competitive and demanding. Recently adolescents have been specializing in sport earlier, indicating more practice and competition, which could ultimately compromise their sleep schedule (20). Adolescent athletes also have obligations that may include social, academic, extracurricular, and job responsibilities (21). Lack of sleep may also be due to technology use, melatonin and hormonal changes, and increased caffeine intake (22, 23). Consequences of insufficient sleep include obesity, mood disorders, and a decrease in educational and physical performance (22, 23).

Most previous research on sleep patterns in athletes examines elite, professional, and collegiate athletes, presenting a gap in the adolescent athlete population. Yet sleep is a strong predictor of injury in adolescent athletes: sleep duration <8 h is associated with a 1.7 times greater risk of injury (24). Therefore, it is important to understand the impact of sports training day on adolescent athletes’ sleep schedules. Adolescent athletes may have different obligations, expectations, and schedules than professional, collegiate, or elite competitors. It is unknown how sports training and competition schedules impact sleep duration and quality in adolescent athletes.

The purpose of this study was to compare sleep patterns in adolescent athletes across training days (i.e., competition, practice, rest). We specifically aimed to determine how sleep was affected by the (1) preparation for a training day (i.e., day before a training day) and (2) response to a training day (i.e., day after a training day). We hypothesized that adolescent athletes would sleep least after a competition day compared with a practice or rest day; and that they would go to bed later and get out of bed later prior to a rest day.

## 2 Methods

### 2.1 Study design

This is a secondary analysis of a study investigating physical activity levels and patterns in adolescent athletes (25); the study was approved by the Marquette University Institutional Review Board (IRB #4495). Data were collected in southeastern Wisconsin between January 2024 and May 2024 and managed in REDCap electronic data capture tools. In person testing, previously described by Street et al. (25), consisted of height, weight, body composition, vertical jump height, activity monitoring, and surveys.

### 2.2 Participants

All participants [*n* = 33 (14 male, 19 female)] were required to be in high school, aged 13–18 years, competing in-season for high school varsity basketball or club volleyball. Written parental informed consent and written adolescent informed assent were obtained prior to data collection. Athletes were excluded if they had an injury or other condition that prevented them from currently participating in full sports activities. Individuals were also excluded if they did not have valid accelerometry data for each type of training day (i.e., competition, practice, and rest). Each participant completed an in-person testing session held at their practice facility. High school basketball athletes traveled to and from their games at local high schools on the same day as the games, which generally occurred in the evening. The club volleyball athletes played in tournaments which were either a long travel distance, or athletes stayed overnight in a different bed.

### 2.3 Accelerometry

Sleep was quantified using an activPAL4 activity monitor (PAL Technologies Ltd, Glasgow, Scotland, United Kingdom), a small, light, and non-invasive triaxial accelerometer. ActivPALs are a valid alternative to the gold-standard polysomnography (26) and chosen for the present study due to feasibility for real-world testing, although activPALs may overestimate time in bed compared to self-reported sleep (27, 28). The activPAL activity monitor was worn on the anterior right thigh approximately 1/3 of the way between the anterior superior iliac spine and the patella. Participants wore the monitor continuously for 14 consecutive days, including when they were practicing and competing. Each participant was taught how to change and properly apply the waterproof film that adhered the monitor to their leg and instructed to only remove the monitor if they were swimming or if their skin became irritated. According to the activPAL 24-hour wear protocol, a valid day is any day with less than four hours of non-wear time (29). Valid days included days in which the activPAL was worn upright and overnight with data recorded. Nights without data were excluded and the first night was excluded because the recording started at midnight. ActivPAL software calculated sleep duration, time in bed start, and time in bed end using the accelerometery data (Table 1).

**Table 1:** Sleep measures and their definitions.

Sleep measure	Definition
Sleep duration	The total time spent primary lying at night
Time in bed start	The time that the participant fell asleep
Time in bed end	The time that the participant woke up
Time in bed midpoint	The time that is halfway between the time they laid down for bed and got up

### 2.4 Sleep diary

After thorough education and familiarization with the task, each participant filled out a daily sleep diary indicating the time they got into bed and the time they got out of bed. Participants also indicated whether the monitor was worn correctly and overnight, leaving notes about non-wear time (e.g., removed to shower or change the waterproof film). Finally, athletes indicated what type of training day (i.e., competition, practice, or rest) they participated in each day. Multisport athletes were instructed to log their highest level of competition, regardless of sport. Training logs were cross-checked with team schedules. A day was excluded if the training day was unknown. Sleep logs were then integrated with the accelerometry data. If bed and wake times were not within 30 min of what was noted on the sleep diary log, researchers used the time in bed adjustment function in the activPAL software to match the sleep log time.

### 2.5 Statistical analysis

Descriptive statistics of participants demographics and other characteristics were conducted using means and standard deviations (SD) or number and percentage (%), as appropriate. The relationship between training day and sleep schedules was assessed using a repeated measure analysis of variance (ANOVA). Assumptions were checked; Mauchly's test of sphericity was tested, and the Greenhouse-Geiser adjustment was used if Mauchly's test of sphericity had a *p*-value of less than 0.05. Only the interaction effect and subsequent *post hoc* analyses were of interest. The analyses were conducted using SPSS Version 29 (IBM, Armonk, New York, United States of America).

## 3 Results

Thirty-nine adolescent athletes enrolled in the parent study, 38 of whom had valid accelerometry-derived sleep data. Of these 38 participants, 33 had all three training days (i.e., competition, practice, and rest day) and were included in the statistical analysis (Table 2). Sleep duration, time in bed start, time in bed end, and time in bed midpoint of the 33 athletes were all measured and reported sequentially below.

**Table 2:** Participant characteristics for the 33 athletes included in this study; values are presented as mean ± standard deviation or number (%).

Participant characteristics (*n* = 33)		Descriptive statistics
Sport	Basketball	10 (30.3%)
	Volleyball	23 (69.7%)
Sex	Male	14 (42.4%)
	Female	19 (57.6%)
Age (yr)		15.6 ± 1.0
Height (cm)		172.7 ± 10.4
Weight (kg)		65.9 ± 10.2
BMI (kg/m^2^)		22.0 ± 2.7
Body fat %		23.9 ± 10.9

### 3.1 Sleep duration

**Figure 1:**
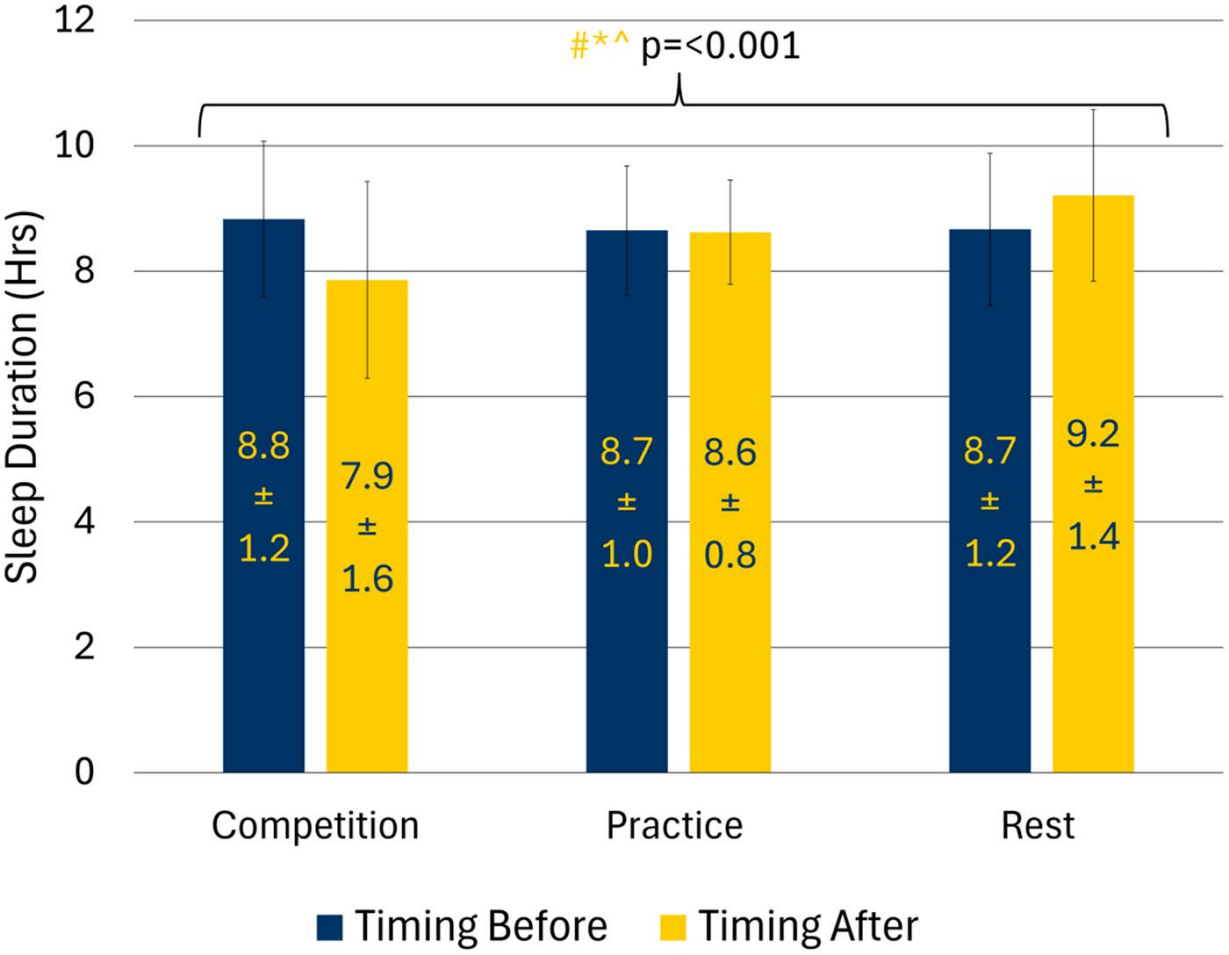
Sleep duration by training day in adolescent athletes; (#: competition vs. rest = < 0.05; *: practice vs. rest = < 0.05; ^: competition vs. practice = < 0.05; navy text indicates significance for comparisons before training day while yellow text indicates significance for comparisons after training day).

There was a significant interaction effect (training day * before and after) for sleep duration. Subsequent *post hoc* analyses identified that comparisons across training day the night after were statistically significant (Figure 1). Sleep duration on the night after a competition was 45.6 min less than the night after a practice day (95% CI: −76.9, −17.0; *p* = 0.006). Athletes slept 81.1 min less on nights after competition days than nights after rest days (95% CI: −118.8, −43.4; *p* < 0.001). Athletes slept 35.5 min less the night after a practice day compared with the night after a rest day (95% CI: −65.9, −5.0; *p* = 0.024). Sleep duration was not significantly different across the nights before training day.

There was also an interaction effect for sleep duration variability (Table 3). Sleep duration nights after competition days were 45.5 min less variable compared to the night after rest days (95% CI: −72.8, −18.3; *p* = 0.002). Nights after practice days were 38.4 min less variable than nights after a rest day (95% CI: −60.2, −16.5; *p* = 0.001). There was no statistically significant difference in variability in sleep duration between the night after competition and the night after practice or across the nights before training day.

**Table 3:** Average time for sleep measures’ variability; values are given as mean ± standard deviation.

Sleep duration variability (hrs)	Competition	Practice	Rest	Interaction *p*-value
Before	1.6 ± 1.3	1.3 ± 1.1	1.5 ± 0.8	**0.046**
After	0.9 ± 0.8	1.1 ± 0.5	1.7 ± 1.0	
Time in bed start variability (hr:min)	Competition	Practice	Rest	Interaction *p*-value
Before	0:56 ± 0:42	0:52 ± 0:42	1:18 ± 0:36	0.846
After	0:53 ± 0:55	0:55 ± 0:31	1:13 ± 0:33	
Time in bed end variability (hr:min)	Competition	Practice	Rest	Interaction *p*-value
Before	1:20 ± 1:04	1:02 ± 1:06	1:50 ± 0:55	0.184
After	0:41 ± 1:01	0:51 ± 0:45	1:38 ± 0:58	
Time in bed midpoint variability (hr:min)	Competition	Practice	Rest	Interaction *p*-value
Before	0:47 ± 0:39	0:52 ± 0:39	1:27 ± 0:33	0.467
After	0:41 ± 0:53	0:47 ± 0:27	1:10 ± 0:34	

*P*-values are bolded if statistically significant (*p* < 0.05).

### 3.2 Time in bed start

**Figure 2:**
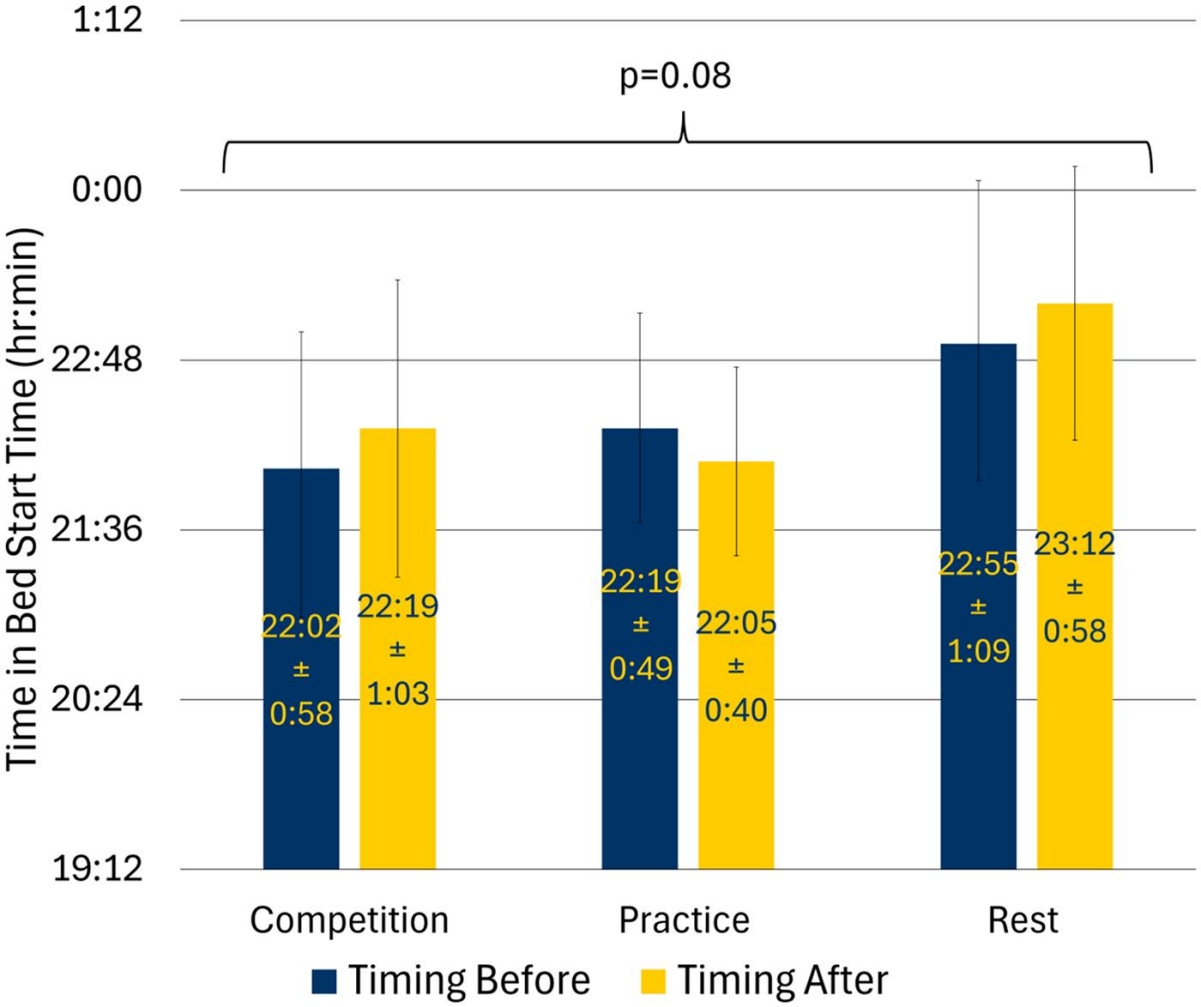
Time in bed start by training day in adolescent athletes; values expressed as average and standard deviations.

The interaction effect between training day and before and after for time in bed start was not statistically significant (*p* = 0.08) (Figure 2). However, *post hoc* comparisons revealed that athletes went to bed later before and after rest days compared to practice or competition days (Supplementary Table 1). The interaction effect between training day and before and after for time in bed start variability was not statistically significant (Table 3).

### 3.3 Time in bed end

**Figure 3:**
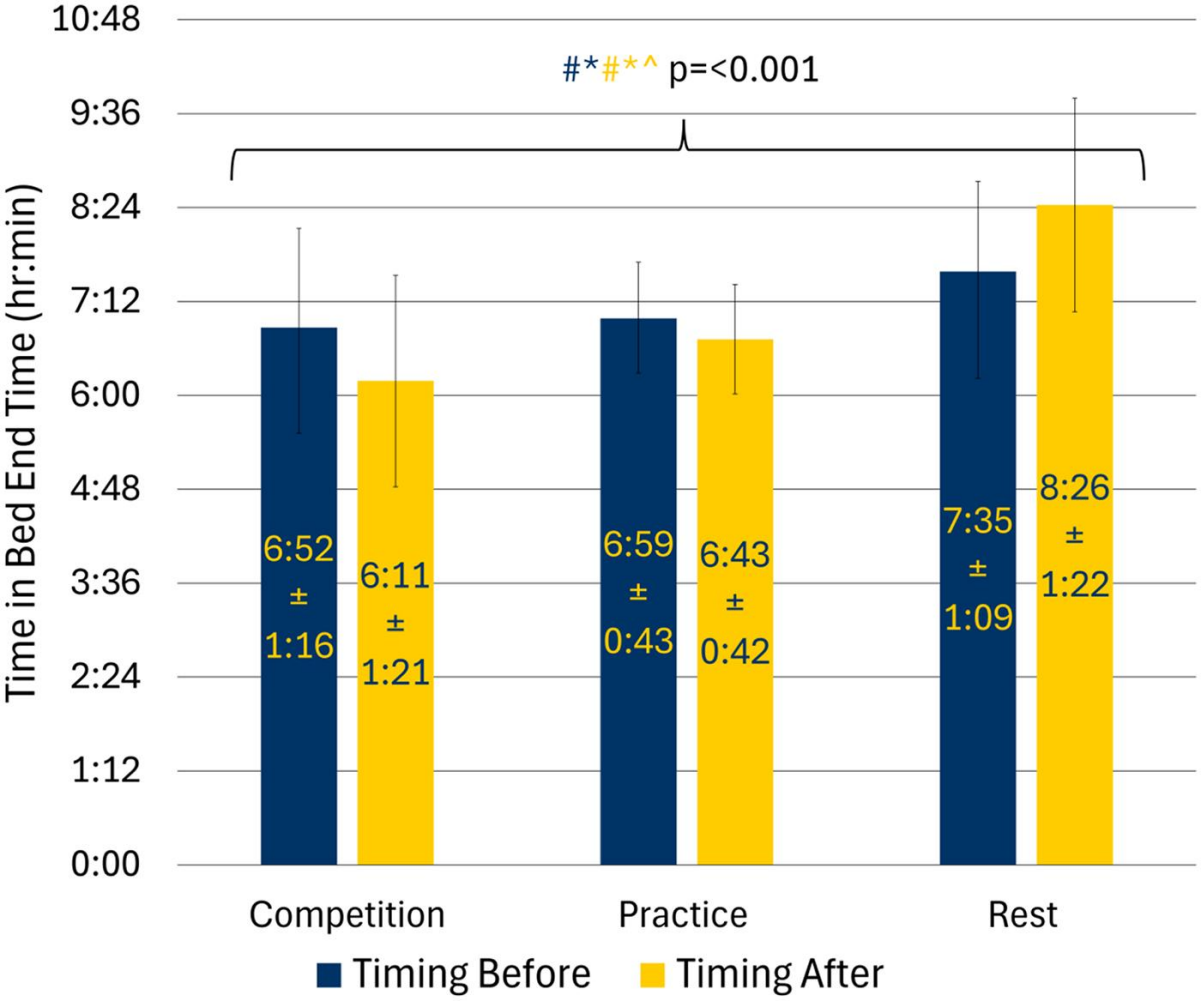
Time in bed end by training day in adolescent athletes; (#: competition vs. rest = < 0.05; *: practice vs. rest = < 0.05; ^: competition vs. practice = < 0.05; Navy text indicates significance for comparisons before training day while yellow text indicates significance for comparisons after training day).

The interaction effect between training day and before and after for time in bed end was statistically significant. Further analyses (Figure 3) showed that the night before competition day athletes woke up 43.5 min earlier than on rest days (95% CI: −80.6, −6.4; *p* = 0.023). They also got out of bed 36.7 min earlier the night before a practice day than rest days (95% CI: −59.6, −13.8; *p* = 0.003). There was no difference in time out of bed between the night before competition day compared with the night before practice day (Supplementary Table 1).

After competition day athletes woke up 31.8 min earlier than after a practice day (95% CI: −60.1, −3.6; *p* = 0.028). Athletes also woke up 134.7 min (2.2 h) earlier after a competition day compared to after a rest day (95% CI: −174.2, −95.2; *p* < 0.001). Athletes woke up earlier after practice days compared to after rest days by 102.9 min (95% CI: −135.5, −70.2; *p* < 0.001). There was no interaction effect for time in bed end variability (Table 3).

### 3.4 Time in bed midpoint

**Figure 4:**
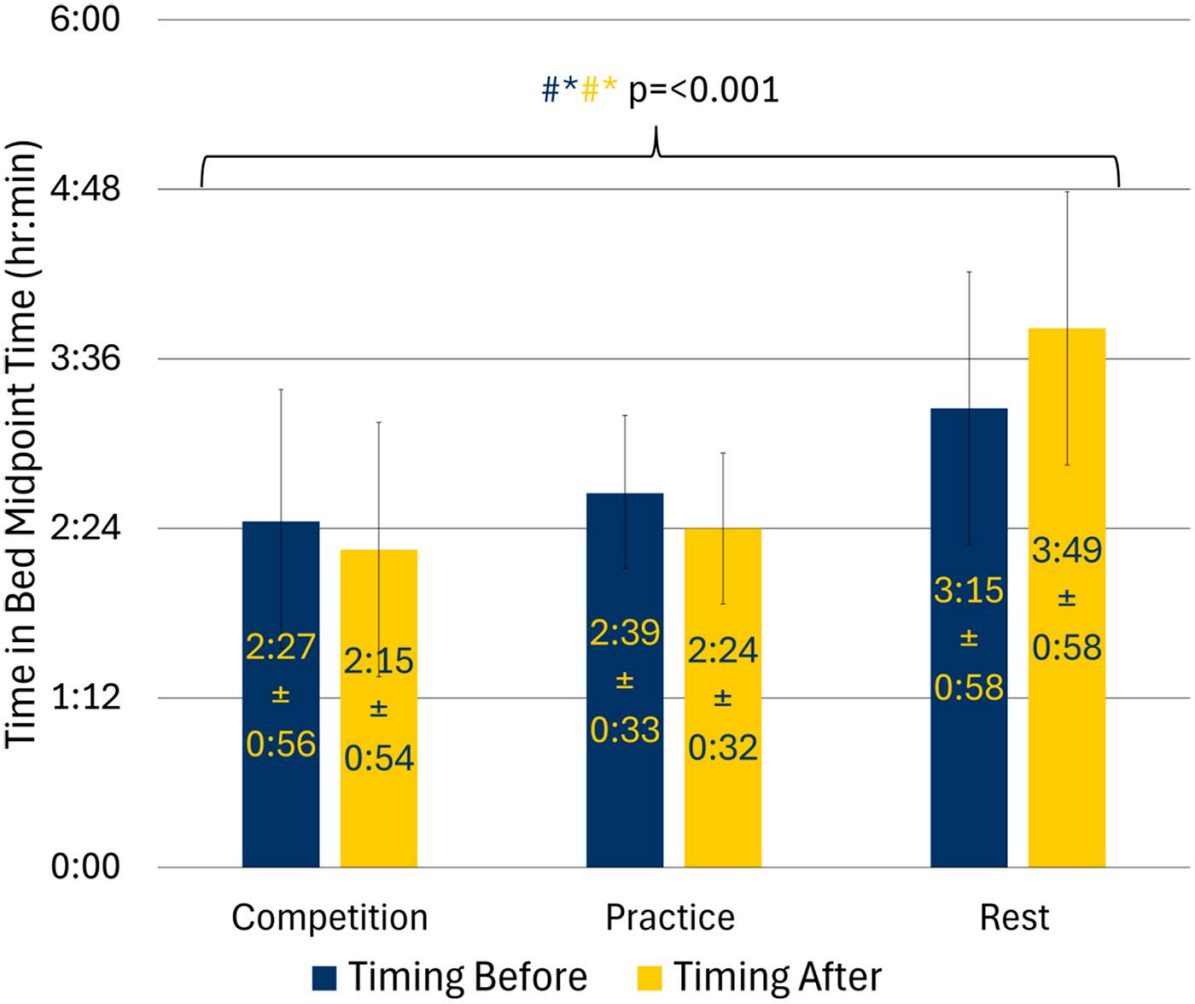
Time in bed midpoint by training day in adolescent athletes; (#: competition vs. rest = < 0.05; *: practice vs. rest = < 0.05; ^: competition vs. practice = < 0.05; navy text indicates significance for comparisons before training day while yellow text indicates significance for comparisons after training day).

There was an interaction effect for the time in bed midpoint (Figure 4). Specifically, the midpoint the night before a competition day was 48.1 min earlier than the night before a rest day (95% CI: −76.5, −19.7; *p* = 0.002). The midpoint the night before a practice day was also earlier than the night before a rest day by 36.1 min (95% CI: −54.4, −17.8; *p* < 0.001). The night before a competition day was not different from the night before a practice day (Supplementary Table 1).

The midpoint the night after a competition day was 94.2 min earlier than the night after a rest day (95% CI: −120.6, −67.8; *p* < 0.001). This trend was also seen when comparing the night after a practice day to that after a rest day. The midpoint the night after a practice day was 85.1 min earlier (95% CI: −107.5, −62.7; *p* < 0.001). The midpoint the night after a competition day was not different compared with the night after practice (Supplementary Table 1). There was no significant interaction between training day and before and after for midpoint variability (Table 3).

## 4 Discussion

Adolescent athletes’ sleep schedules change according to their sports training day (i.e., competition, practice, and rest days). Our first hypothesis, that athletes would sleep less after a competition, was supported. Our findings suggest that adolescent athletes do not have a regular sleep schedule which may negatively affect their overall health and impair their performance given the substantial prior research literature expounding the benefits of sleep (1–3, 6–10, 22, 23). Our second hypothesis, that athletes would go to bed later and get out of bed later after a rest day, was largely supported. Our findings indicate that athletes wake up later after a rest day and tend to go to bed later on nights before a rest day.

Consistent with prior literature, adolescent athletes sleep less the day after a competition (16, 17). There are many factors that may explain this phenomenon, including higher levels of inflammatory cytokines from intensive exercise (16), increased circulating cortisol (30), sympathetic hyperactivity (31), and elevated core body temperature (32). These are all metabolic markers in the body that increase with hard training or competition. They cause arousal and may be the reason why sleep duration is shorter after competition. Not only does competition itself affect sleep, but the travel associated with competition may also affect sleep duration (17). Late night or early morning travel to and from games may disrupt the athlete's sleep schedule which is corroborated by prior research (16, 17).

Sleep duration variability is highest the night after rest days. This finding indicates that there is inconsistency in adolescent athletes’ sleep schedules the night after a rest day. Rest days may be less structured, as athletes are not participating in practice or games and they have increased sedentary behavior (25), thereby allowing adolescent athletes more freedom in their schedules including their sleep on these nights. Athletes slept longer on these nights, perhaps to ‘catch up’ on sleep. Without prior commitments, athletes could sleep later and longer. Deep sleep is important for athletes, helping with physical and mental recovery as well as illness and injury prevention (33). Although the sleep duration average indicates the athletes sleep more before and after rest days, the greater variability may indicate that there may be some nights athletes are getting less sleep while other nights they are getting much more.

Time in bed start tended to be later before and after rest days compared with competition and practice days. Sargent et al. found that some athletes have tried going to bed earlier in preparation for an early morning training wake-up call, however they are often unable to fall asleep earlier (14). A possible explanation for why athletes may go to bed later on rest days is that they have more time to engage in social events or complete other activities in their free time on rest days. Athletes may recognize that they can stay up later because they do not have obligations the next day. This may cause athletes to use screens for longer periods of time before bed. Screens, including but not limited to phones and TVs, increase sleep problems in adolescent athletes (34). Some of these problems include later time in bed start, sleep disturbances, and overall bedtime resistance (34). It is important that athletes maintain sleep schedule consistency because going to bed later can affect performance measures in athletes (35).

Time in bed end was earliest on competition days compared to practice and rest days regardless of being before or after the training day. This may be because of early competition times or early start of school. Roberts et al. identified that as practice is moved earlier in the morning sleep duration becomes shorter (16). They also identified this same trend in society, looking at sleep duration in adults with professions that had an early start time (16). Although some of the adolescent athletes in our study competed in the afternoon or night, this idea that sleep duration may be affected by time in bed end could be transferred to students waking up early for school or games. Another explanation for early wake times is that athletes may wake earlier because they are nervous about the upcoming competition. Juliff reported that 64% of athletes aged 16–47 years stated they slept worse the night before a competition and 27% of these athletes reported their lack of sleep was due to waking up early (36). Most of the athletes also reported their decrease in sleep was due to thoughts about the competition (36).

Time in bed midpoint is the halfway point between going to bed and waking up and it is commonly reported in literature as a highly generalizable sleep variable. The latest midpoint was the day after a rest day while the earliest was the day after competition. Consistent with prior literature, this shows the variability overall in athletes sleep schedules throughout the week and athletic season (12). There are many factors that can play into these differences in sleep quantity and timing including physical condition and mental condition, training and travel schedules, and overall health.

### 4.1 Limitations

There are limitations to consider when interpreting the findings of this study. The sample size was small and from a single geographic region due to feasibility of a pilot study, although each athlete served as their own control and this approach strengthens internal consistency. To capture all participants in season we recruited only volleyball and basketball athletes and thus the findings may not apply to athletes in other sports with different practice and competition schedules. We were unable to capture sleep efficiency (i.e., when athletes got in bed vs. when they fell asleep) or nighttime awakenings which could also be influenced by training day status. Furthermore, we did not directly assess performance or the impact of sleep on performance, although considerable prior research has shown well-established health, performance, and recovery benefits of sleep. Although activPAL is the best option for our type of study it may overestimate time in bed which we minimized by crosschecking with the sleep diaries.

### 4.2 Future directions

Future research should build off this pilot study by examining whether the observed findings are consistent in a larger and more heterogeneous sample of adolescent athletes across many sports and geographic regions. Future studies could also investigate how physiological markers, training load, and travel schedules affect sleep in adolescent athletes and relate these factors and sleep measures to predict performance outcomes in athletes. These findings may inform potential interventions regarding sleep, ultimately helping to prevent injuries and improve overall performance in adolescent athletes.

## 5 Conclusion

There is an association between sports training day and adolescent athlete's sleep schedules. Athletes slept least the day after competition. The time athletes went to bed tended to be later before and after rest days. They woke up latest the morning after a rest day. These findings may provide insight into athletes’ recovery from competitions and readiness to train or compete and help coaches, parents, and healthcare providers optimize sleep habits in young athletes.

## Data Availability

The original contributions presented in the study are included in the article/Supplementary Material, further inquiries can be directed to the corresponding author.
