# Supplementary material for: Sport training day affects adolescent athletes’ sleep schedules
Source: Front Sports Act Living. 2026 Jan 27;7:1731173. doi: 10.3389/fspor.2025.1731173 (PMC12887853; doi:10.3389/fspor.2025.1731173)
Supplement: Supplementary file 1 [file Table1.docx]

Supplementary Material

# Supplementary Tables

**Supplementary Table 1.** Post hoc analyses for sleep measures by training day in adolescent athletes. P-values are **bolded** if statistically significant (p<0.05) after a significant interaction effect (training day * before and after).

|  | | | | **Mean Difference** | **Standard Error** | **P-Value** | **95% CI Lower** | **95% CI Upper** |
| --- | --- | --- | --- | --- | --- | --- | --- | --- |
| **Sleep Duration (mins)** | Before | Competition | Practice | 10.38 | 14.34 | 0.473 | -18.78 | 39.6 |
|  |  | Competition | Rest | 9.18 | 16.68 | 0.586 | -24.78 | 43.14 |
|  |  | Practice | Rest | -1.2 | 13.38 | 0.927 | -28.44 | 25.98 |
|  | After | Competition | Practice | -45.6 | 15.36 | **0.006** | -76.92 | -16.98 |
|  |  | Competition | Rest | -81.12 | 18.54 | **<0.001** | -118.8 | -43.38 |
|  |  | Practice | Rest | -35.52 | 14.94 | **0.024** | -65.94 | -5.04 |
| **Time in Bed Start (mins)** | Before | Competition | Practice | -17.24 | 13.965 | 0.226 | -45.686 | 11.206 |
|  |  | Competition | Rest | -52.705 | 14.013 | <0.001 | -81.248 | -24.161 |
|  |  | Practice | Rest | -35.465 | 11.143 | 0.003 | -58.163 | -12.767 |
|  | After | Competition | Practice | 13.74 | 10.216 | 0.188 | -7.069 | 34.55 |
|  |  | Competition | Rest | -53.625 | 11.48 | <0.001 | -77.01 | -30.241 |
|  |  | Practice | Rest | -67.366 | 9.836 | <0.001 | -87.402 | -47.33 |
| **Time in Bed End (mins)** | Before | Competition | Practice | -6.839 | 16.55 | 0.682 | -40.55 | 26.871 |
|  |  | Competition | Rest | -43.537 | 18.217 | **0.023** | -80.644 | -6.43 |
|  |  | Practice | Rest | -36.698 | 11.254 | **0.003** | -59.622 | -13.773 |
|  | After | Competition | Practice | -31.844 | 13.867 | **0.028** | -60.091 | -3.598 |
|  |  | Competition | Rest | -134.711 | 19.378 | **<0.001** | -174.183 | -95.238 |
|  |  | Practice | Rest | -102.866 | 16.015 | **<0.001** | -135.488 | -70.245 |
| **Time in Bed Midpoint (mins)** | Before | Competition | Practice | -12.04 | 13.532 | 0.38 | -39.603 | 15.524 |
|  |  | Competition | Rest | -48.121 | 13.952 | **0.002** | -76.54 | -19.701 |
|  |  | Practice | Rest | -36.081 | 8.991 | **<0.001** | -54.395 | -17.767 |
|  | After | Competition | Practice | -9.052 | 9.439 | 0.345 | -28.279 | 10.175 |
|  |  | Competition | Rest | -94.168 | 12.957 | **<0.001** | -120.561 | -67.775 |
|  |  | Practice | Rest | -85.116 | 10.988 | **<0.001** | -107.498 | -62.734 |

**Supplementary Table 2.** Post hoc analyses for sleep measures variability by training day in adolescent athletes. P-values are **bolded** if statistically significant (p<0.05) after a significant interaction effect (training day * before and after).

|  | | | | **Mean Difference** | **Standard Error** | **P-Value** | **95% CI Lower** | **95% CI Upper** |
| --- | --- | --- | --- | --- | --- | --- | --- | --- |
| **Time in Bed Duration Variability (mins)** | Before | Competition | Practice | 19.26 | 16.62 | 0.255 | -14.7 | 53.28 |
|  |  | Competition | Rest | 4.44 | 15 | 0.769 | -26.28 | 35.22 |
|  |  | Practice | Rest | -14.82 | 10.56 | 0.171 | -36.42 | 6.78 |
|  | After | Competition | Practice | -7.14 | 9.54 | 0.46 | -26.76 | 12.42 |
|  |  | Competition | Rest | -45.54 | 13.32 | **0.002** | -72.78 | -18.3 |
|  |  | Practice | Rest | -38.4 | 10.68 | **0.001** | -60.24 | -16.5 |
| **Time in Bed Start Variability (mins)** | Before | Competition | Practice | 3.076 | 10.651 | 0.775 | -20.408 | 24.893 |
|  |  | Competition | Rest | -22.385 | 9.189 | 0.021 | -41.209 | -3.562 |
|  |  | Practice | Rest | -25.461 | 9.853 | 0.015 | -45.645 | -5.277 |
|  | After | Competition | Practice | -2.292 | 10.441 | 0.828 | -23.68 | 19.097 |
|  |  | Competition | Rest | -20.42 | 11.472 | 0.086 | -43.969 | 3.079 |
|  |  | Practice | Rest | -18.128 | 7.507 | 0.023 | -33.506 | -2.75 |
| **Time in Bed End Variability (mins)** | Before | Competition | Practice | 17.755 | 18.665 | 0.35 | -20.478 | 55.987 |
|  |  | Competition | Rest | -30.202 | 12.99 | 0.028 | -56.811 | -3.594 |
|  |  | Practice | Rest | -47.957 | 11.492 | <0.001 | -71.498 | -24.417 |
|  | After | Competition | Practice | -9.548 | 12.997 | 0.469 | -36.171 | 17.076 |
|  |  | Competition | Rest | -56.536 | 10.801 | <0.001 | -78.661 | -34.411 |
|  |  | Practice | Rest | -46.989 | 11.377 | <0.001 | -70.294 | -23.683 |
| **Time in Bed Midpoint Variability (mins)** | Before | Competition | Practice | -4.913 | 9.329 | 0.603 | -24.024 | 14.197 |
|  |  | Competition | Rest | -39.189 | 9.167 | <0.001 | -57.966 | -20.412 |
|  |  | Practice | Rest | -34.276 | 6.881 | <0.001 | -48.372 | -20.18 |
|  | After | Competition | Practice | -6.355 | 10.417 | 0.547 | -27.693 | 14.983 |
|  |  | Competition | Rest | -29.272 | 8.391 | 0.002 | -46.46 | -12.083 |
|  |  | Practice | Rest | -22.917 | 5.726 | <0.001 | -34.645 | -11.188 |
